# Public perceptions of emergency decontamination: Effects of intervention type and responder management strategy during a focus group study

**DOI:** 10.1371/journal.pone.0195922

**Published:** 2018-04-13

**Authors:** Holly Carter, Dale Weston, Naomi Betts, Simon Wilkinson, Richard Amlôt

**Affiliations:** 1 Emergency Response Department Science & Technology, Public Health England, Porton Down, Salisbury, Wiltshire, United Kingdom; 2 Medical Toxicology Centre, Newcastle University, Wolfson Building, Claremont Place, Newcastle upon Tyne, United Kingdom; Uniformed Services University of the Health Sciences, UNITED STATES

## Abstract

In the event of an incident involving the release of a hazardous chemical, first responders may decide to initiate emergency decontamination in order to remove any contaminant from affected casualties. Recent initiatives such as the UK Home Office-led Initial Operational Response Programme have introduced new evidence-based decontamination protocols that reduce the time taken to initiate the decontamination process, including an increased emphasis on rapidly removing contaminated clothing (disrobe), and the use of improvised dry decontamination methods. The current study used a series of focus groups to examine public perceptions of different decontamination interventions and responder management strategies. Results revealed that a decontamination shower was perceived to be more effective than dry decontamination methods and that a management strategy that included effective responder communication resulted in increased willingness to comply with the need for decontamination. This study demonstrates that public understanding and acceptance of novel decontamination methods such as dry decontamination may present additional challenges for first responders. Increased emphasis on effective communication during decontamination is needed. Furthermore, provision of information during the focus group study resulted in an increase in participants’ knowledge and confidence in taking recommended decontamination actions, which was maintained three months after the study. The longitudinal nature of these effects suggest that it may be possible to increase public awareness about actions to take during chemical incidents by developing pre-incident public education; however, further research is needed to examine this more fully.

## Introduction

In the event of an incident involving the release of a hazardous chemical, emergency responders may decide to initiate emergency decontamination. This involves the use of physical methods (traditionally, showering in a specialist mass decontamination unit [1] to remove the contaminant from affected casualties. There are two key issues when it comes to mass decontamination. First, specialist equipment may not be immediately available, and this lack of availability could result in delays in initiating emergency decontamination. Research increasingly indicates that speed will be essential during decontamination, and that it may not be appropriate to wait for specialist equipment before initiating decontamination [2]. Second, there has been a lack of focus on understanding the psychosocial aspects involved in managing mass decontamination. For example, factors affecting whether members of the public will comply with decontamination, how anxious members of the public are likely to be, and whether members of the public are likely to cooperate with responders and each other.

The way in which members of the public perceive decontamination interventions can impact on the success of the decontamination process, with effective management resulting in more positive public perceptions of decontamination, and hence more positive outcomes [3–5]. An effective management strategy is one which includes practical information, health-focused explanations about the need for decontamination, and respect for public needs, such as needs for privacy [4].

Where communication is effective and public needs are respected, this results in improved public compliance and cooperation during decontamination, due to increased perceptions of responder legitimacy [3–5]. The available research has focused on understanding factors affecting public experiences and perceptions during incidents involving mass decontamination in an MD1 unit [3–5]. However, the nature of emergency response involving decontamination is changing, and as such, there is a need to understand more about public perceptions of other types of decontamination interventions. One such possible intervention is dry decontamination (e.g. using available absorbent materials to remove a contaminant from the skin); this is discussed in more detail below.

A recent UK Home Office initiative, the Initial Operational Response Programme (IOR) [6], outlines procedures and guidance to enable non-specialist first responders to initiate decontamination rapidly, prior to the arrival of specialist teams and equipment. IOR specifies several key stages in the management of a chemical incident, including: evacuation; removal of contaminated clothing (disrobe); and dry decontamination. IOR guidance suggests that, unless the suspected contaminant is a caustic chemical or particulate contaminant, dry decontamination should be carried out as soon as possible. The IOR programme is therefore designed to allow decontamination procedures to be initiated as rapidly as possible, without the need to wait for specialist teams and equipment.

Another way in which decontamination may be initiated more rapidly is by providing pre-incident public education about how to undergo different forms of decontamination [7]. Evidence suggests that providing pre-incident public education about actions to take during hazardous chemical incidents could reduce the time needed for people to take initial actions (e.g. evacuation, disrobe) [8–10]. Such information would therefore enable members of the public to take actions to reduce their own risk, prior to the arrival of any emergency responders; this increased speed in initiating decontamination could save lives [2].

There is currently very limited evidence relating to perceived public acceptability of employing dry decontamination instead of, or in addition to, wet decontamination, or whether members of the public would be happy to comply with a dry decontamination process. Available evidence suggests that members of the public may not feel clean after undergoing dry decontamination [11], and this could affect their willingness to comply with the process, and also result in a desire to seek further treatment. It is important to understand any factors which affect the perceived acceptability of different dry and wet decontamination interventions.

The current research involved carrying out a series of focus groups in which participants were presented with a hypothetical scenario involving the release of a hazardous chemical, and asked to visualise that they had been involved in the incident described. Each group was then presented with one of four different descriptions of a hypothetical decontamination intervention. There were four main aims: 1) to examine the perceived acceptability of different decontamination interventions (e.g. a decontamination shower, dry decontamination), and why some interventions may be perceived as more acceptable than others; 2) to examine the effect of different responder management strategies (e.g. those that emphasise respect and the provision of information, versus control-focused strategies where information is deliberately limited) on public perceptions of decontamination interventions, and the impact of public perceptions on likely public behaviour; 3)to provide an insight into how taking part in a focus group relating to incidents involving decontamination can improve public understanding of and preparedness for real incidents of this type, both in the immediate and longer terms; and 4) to use information collected from focus groups to inform the development of decontamination guidance and protocols for emergency responders.

## Method

This study was approved by the King’s College London Psychiatry, Nursing and Midwifery Research Ethics Subcommittee. Approval number: HR-15/16-1909.

### Design

Within a focus group study, a 2 x 2 x 3 mixed design was used to present the decontamination intervention types and management strategies, and to explore whether participation in the study affected participants knowledge of decontamination over time. The between-subjects component had two factors, each with two levels: decontamination intervention type (dry decontamination followed by wet decontamination, and wet decontamination alone); and type of management strategy (respect management strategy i.e. high information, and control management strategy i.e. low information). The within-subjects component had one factor (time) with three levels: Time 1, before receiving the intervention; Time 2, after taking part in the focus group; Time 3, 3-months post-focus group.

### Participants

Participants were a representative sample of 62 members of the public from the London area, recruited via a market research recruitment company. Inclusion criteria for participation in this study were that participants must be aged 18–65, and must be able to speak fluent English. Thirty volunteers (48%) were male and 32 (52%) were female. The proportion of different genders, ethnicities, and ages was representative of the wider London population. Participants opted-in to the study, and no participants dropped out after agreeing to take part.

### Materials

#### Scenario

A scenario was developed which described an incident involving the release of a non-caustic, liquid contaminant (S1 Text). Participants were asked to read the scenario and visualise that they had been involved in the incident described, before being asked to complete self-report questionnaires and take part in focus group discussions.

#### Interventions

Four different responder interventions were developed. The different interventions varied based on the decontamination method and responder management strategy described. The four different interventions were: 1) dry decontamination, followed by wet decontamination/respect management; 2) dry decontamination, followed by wet decontamination/control management; 3) wet decontamination alone/respect management; 4) wet decontamination alone/control management. See S2 Text for a copy of the four different interventions.

#### Pre-focus group questionnaire

The pre-focus group questionnaire contained scales relating to: participants’ existing knowledge and confidence in taking protective decontamination actions in the event of a CBRN incident (e.g. “If a real incident of this type were to occur, I would know what actions to take to protect myself”) (4 items); perceptions of legitimacy of responder actions (e.g. “I think that the emergency services would behave in a respectful way when managing this type of incident”) (2 items); and perceptions of social support offered by others affected (e.g. “If this were a real incident, I would expect to receive help from other members of the public who were involved”) (3 items). All scales had good reliability (α > .7). See S3 Text for the full pre-focus group questionnaire.

#### Post-focus group questionnaire

The post-focus group questionnaire contained similar scales to those contained in the pre-focus group questionnaire, including: participants’ existing knowledge and confidence in taking protective decontamination actions in the event of a CBRN incident (4 items); perceptions of legitimacy of responder actions (2 items, α); and perceptions of social support offered by others affected (3 items). The post-focus group questionnaire also contained individual items relating to how participants would feel when undergoing decontamination (e.g. how comfortable they would feel, and how embarrassed they would feel), and participants’ perceptions of the decontamination method described (e.g. how easy decontamination would be to undertake, and how effective the decontamination method would be). All scales had good reliability (α > .7). Those whose scenarios contained both dry decontamination and wet decontamination were asked these questions relating to both methods, while those whose scenarios did not contain dry decontamination were asked about only wet decontamination. Individual items also measured participants’ willingness to comply with the need for decontamination, participants’ expectations of their anxiety levels during such an incident, and participants’ likelihood to seek further treatment following decontamination. See S4 Text for the full post-focus group questionnaire.

#### 3-month follow-up questionnaire

The 3-month follow-up questionnaire contained the scale relating to participants’ existing knowledge and confidence in taking protective decontamination actions in the event of a CBRN incident (4 items). The scale had good reliability (α > .7). This questionnaire was designed to allow a comparison between participants’ knowledge and confidence prior to taking part in the focus group, and their knowledge and confidence immediately after taking part in the focus group, with their knowledge and confidence 3 months later. See S5 Text for the full 3-month follow-up questionnaire.

#### Discussion guide

The discussion guide contained similar questions to those on the pre- and post-focus group questionnaires (see S6 Text).

### Procedure

Participants arrived and were given an information sheet (S7 Text), before being asked to sign a consent form (S8 Text). They were then asked to read the scenario, before being asked to complete a pre-focus group questionnaire (prior to taking part in any focus group discussions). Participants then took part in a focus group discussion about the scenario, before being asked to read one of the four decontamination intervention injects, and taking part in a further focus group discussion about the intervention. They were then asked to complete the post-focus group questionnaire, before receiving a debrief statement. In total, each focus group lasted for approximately 2 hours. Participants were informed that they could request a copy of the findings from the study if they wished. A follow-up questionnaire was sent to participants 3 months after their having taken part in a focus group, to examine to what extent knowledge and confidence gained through taking part in the focus group was retained at a 3-month interval.

### Analysis

#### Focus group data

Focus groups were recorded and transcribed. Data were analysed using the framework approach, a type of thematic analysis which is often used in research which has implications for policy [12]. A thematic framework was identified, based on the aims of the study, and the relevant issues highlighted in previously published literature. Data were categorised into three broad themes of interest, each of which was then divided into relevant sub-themes. Each passage within the data was then coded into one or more of the relevant themes. By the end of analysis and coding no new themes emerged from the data, and thus data saturation had been reached. The lead researcher coded all focus groups, and a sub-section of data (3 focus groups each) was also coded by two other researchers. Themes identified were consistent across coders. See Table 1 for the themes and sub-themes identified.

**Table 1:** Themes and sub-themes identified during focus group analysis.

Themes	Sub-themes
Perceived acceptability of and confidence in different decontamination interventions	perceived acceptability of decontamination using blue roll
	perceived acceptability of undergoing a decontamination shower
Perceptions of different responder management strategies	perceived effectiveness of communication provided
	perceptions of emergency responders
	likely behavioural outcomes
	likely psychological outcomes
Emerging factors	the importance of considering the needs of vulnerable individuals
	perceptions of the situation as a life and death
	the possibility of developing pre-incident public education

#### Questionnaire data

Questionnaire data was analysed using SPSS 22.0. A one-way within-subjects ANOVA was carried out to compare knowledge and confidence in taking protective actions between Time 1, Time 2, and Time 3. Two-way ANOVAs were carried out to examine the effect of communication condition and decontamination condition on variables at Time 2; this was the case for any variables which were completed by all participants (i.e. items relating to wet decontamination). One-way ANOVAs were carried out to examine the effect of communication condition on variables at Time 2; this was the case for any variables which were completed by only those participants whose scenario included dry decontamination.

## Results

The results are presented by theme and sub-theme. A letter and a number appear in brackets following each quote, for example (A1). The letter indicates which condition each participant was in (A = control management strategy, wet decontamination only, B = control management strategy, dry decontamination and wet decontamination, C = respect management strategy, wet decontamination only, D = respect management strategy, dry decontamination and wet decontamination) and the number illustrates which focus group the participant was in within each condition (each condition included three separate focus groups).

### Qualitative results

#### Perceived acceptability of using blue roll

Those in the control management strategy group were almost unanimous in their negative views about blue roll. These views included a suggestion that blue roll wouldn’t be effective, for example “If it’s dried, it’s not, the paper towels ain’t going to do anything, is it” (B3), that it is a pointless or inappropriate intervention, “it would be pointless to use blue roll to decontaminate yourself, how can you decontaminate yourself with tissue paper?” (B2), or even that it could potentially make the situation worse, “say if you did wipe it off you and then like you’re wiping it, you could spread it onto other parts of your body” (B1).

Those in the respect management strategy group expressed more mixed views about blue roll. As in the control management strategy group, some suggested that blue roll was a pointless intervention, for example”that blue roll just doesn’t seem to do anything” (D2). However, some people suggested that blue roll was unlikely to do any harm, and so they would be happy to undergo this as an initial intervention, “I don’t know just anything that I thought wasn’t going to be a detriment, I’d probably do it” (D2). Others believed that blue roll was mainly used to keep people occupied, and thus calmer, while they waited for the shower, but felt that overall this would have a positive effect, “the shower’s really what they need to do to you, but they got to get you to feel like you are doing something” (B1).

There was therefore little difference between the control management strategy group and the respect management strategy group, in terms of their beliefs about the efficacy of blue roll as a decontamination intervention. However, there was a difference in that views about the use of blue roll were generally more positive in the respect management condition, and participants in this condition expressed greater willingness to comply with the use of blue roll. It is possible that this is due to the greater amount of information provided about the use of blue roll in the respect management strategy condition, this idea is discussed in more detail in the perceptions of responder management strategies section below.

#### Perceived acceptability of a decontamination shower

Views about the efficacy of the shower were broadly consistent across all groups, regardless of whether or not they were given blue roll first. Views on shower efficacy also did not seem to be affected by the amount of information received. Generally, groups were evenly split between those who felt that the decontamination shower is an appropriate first step, for example “the shower yes, definitely, that is a standard way of doing things” (B2), and those who felt that the showering process seemed too basic “I don’t know if I would feel that would be enough though, just water” (A1), or unrealistic, “I think that’s not really realistic, the showering” (D3).

In the two groups whose interventions included blue roll and a decontamination shower (conditions 2 and 4), the shower was consistently perceived to be more effective and acceptable than blue roll. Comments comparing decontamination with blue roll to a decontamination shower included, “the more I think about it the more I think I’d been fobbed off until I got in the shower” (B1), “the blue roll is not my saviour, just get me in the shower!” (B3) “You’re going into a shower anyway, you’re going to have a towel after that, so the blue roll seems unnecessary at the time” (D2). However, willingness to comply with using the blue roll was higher in the condition that received the respect management strategy when compared to the condition that received the control management strategy; this finding is discussed in more detail in the section on perceptions of different responder management strategies, below.

A consistent finding across all groups was that those affected would want to seek further treatment following decontamination, for example, “I probably wouldn’t expect them to let you just have a shower and go” (C1). Suggestions for the type of further treatment people would want included: physical check up at the scene, “a medical team would be good to assess how bad you are” (B2); a visit to hospital or GP, “after I’ve had a shower, I’d wanna be taken to hospital” (C2); follow up phone calls “You’d kind of hope that they’d set up some sort of helpline for anyone that was involved” (B3); and follow up information “I would expect someone to be there talking me through the steps of aftercare and who I need to see, when I’m getting checked up again and if there’s anything I need to do at home” (C3).

#### Perceived effectiveness of communication provided–control communication

Those in the two groups who received the control communication strategy were almost unanimous in their feeling that the information they had received was not sufficient. Comments from participants in these two conditions included: “they’re not telling what will happen if you don’t go in there” (A3), “no the level of information here is not enough, there’s no information” (B2).

In the control communication strategy conditions, participants reported that the additional information they needed included: what the chemical is, “I’d want to know what the chemical was, and have they had experience of that in the past” (A1); the effectiveness of decontamination, “I’d wanna know if water was sufficient to decontaminate in the first place” (B2); follow up information “I’d need to go home with a card with an emergency contact number” (B3), “if they’re telling you to leave, I’d quite like some information on whether you are going to contaminate your loved ones” (A2); and practical information about the decontamination process, “I’d think maybe they would need to tell you how long you need to remain in the shower” (A3), “you would need instructions at every point, how to do this, what to do next” (B1).

Suggestions for why information was important included: it would help to keep people calm and reduce anxiety, “the constant flow of information will keep people calm, you leave them with silences and the rot will set in” (B1); it would help to increase compliance, “if someone’s telling you a reason why you need to stay there you’re more inclined to do it” (A2), “you have to tell people, keep them informed of what’s happening, what’s going to happen next, and then people will be more responsive” (A3); it will reduce confusion and make sure the process is carried out effectively, “[without information] someone might just go in and then walk straight out again whereas that’s not going to get all the contamination off you” (A2); and it will foster a good relationship between emergency responders and members of the public, “the more information you have [the more you feel] that maybe you’re part of it, rather than just a number that’s being herded like a sheep through a pen” (A3).

#### Perceived effectiveness of communication provided–respect communication

Those in the two respect communication strategy conditions were more mixed in their views about the information provided than were those in the two control strategy conditions. Some participants felt that the information provided would not be sufficient, for example “I think you would probably have to give people a little bit more information to reassure them” (D3). However, a similar number also expressed that they thought the information that was provided would be sufficient, “it’s really good that they’re explaining why you take the top layer of your clothes off to get rid of 80 to 90 per cent” (C3).

Additional information needs reported by those in the respect communication strategy conditions were similar to those reported in the two control communication strategy conditions, and included: a need to know what the chemical is “I think I’d want to know what it was to be quite honest” (D2),; explain the importance of undergoing decontamination, “I think they should tell you before you go through the shower, that after you go through the shower and wipe yourself you’re going to be clean, instead of telling you at the end” (D2); practical information about how to undergo decontamination, “don’t touch your eyes with it [blue roll], stuff like…just some safe…safe way to use it” (D1); confirmation that decontamination has been effective, “I’d wanna know that it, I was ok to go home more or less” (C2), follow up information, “you definitely want information to know what to do if you had any other symptoms” (D3) “if there was a leaflet and a packet a package or something an aftercare […] then you would feel a little bit more, like right ok that’s what happened” (D3).

Again, reasons given by the participants on why information is important were similar to those given in the control communication conditions, and included: provision of information would increase compliance, “let people understand why things are happening […] then people [will be] more likely to, to go along with it” (C3), “until they’ve explained to me what chemical it was, I still wouldn’t feel ok to go home” (D1); provision of information would increase confidence in emergency responders, “you’ve got to read between the lines as an adult, and if they’re not answering you, they don’t know” (C2); and provision of information would reduce anxiety, “I think to soothe that worry the emergency services would have to explain what’s about to happen” (C3), “I think as much information as possible: helplines, leaflets…whatever you can get your hands on. Will just…ease the pain” (D1).

#### Perceptions of emergency responders–control communication

In both of the control communication conditions, most participants expressed a belief that emergency responders would behave in a positive way during mass decontamination. Comments included: “they’d be very respectful and…do exactly as they’ve been trained” (A1), “I expect them to be calm and respectful” (A2), “I would like to think they’d behave professionally” (B2). However, some participants expressed more negative expectations of responder behaviour, such as, “empathy for the situation from the people that the emergency services is pretty poor ‘cause it’s kind of like a glorified sheep dip basically” (A2), “I don’t think it’ll be like the sharpest operation that ever happened” (B3).

#### Perception of emergency responders–respect communication

As with the two control communication conditions, the majority of participants in the two respect communication conditions reported positive expectations of responder behaviour: “I’d expect them to be quite firm with you, organised, assertive, not rude” (C2), “they’re very well trained…I have quite a lot of faith in them” (D1). Again, there were a few participants who expressed more negative expectations of emergency responders, which included: “I think we put too much faith sometimes in the services because I think at the train station they can’t deal with a cancellation at Waterloo, so…if anything else happened God help us!” (D1), “that level of respect that you might want ordinarily be afforded is removed somewhat, to the point where you know even just maybe you know you end up being physically manhandled” (D2).

#### Likely behavioural outcomes–control communication

Two main behavioural outcomes were identified–compliance and willingness to help others. Among those in the shower-only condition, willingness to comply with the need for a decontamination shower was mixed, with some participants stating that they would be willing to undergo a decontamination shower, and others saying that they would not. Among those in the blue roll condition and shower condition, people were more likely to say that they would comply with a decontamination shower, but were mixed in their views as to whether they would comply with using blue roll to carry out initial dry decontamination. It is possible that this is because the decontamination shower was perceived to be more effective, and therefore acceptable, in comparison to using blue roll, and that this is why views on the decontamination shower were more positive in the group who were also asked to use blue roll; this will be discussed in more detail in the discussion.

Factors which were suggested to increase willingness to comply included: providing sufficient information about the need for decontamination, for example, “the best way to deal with it rather than um, sort of herd everybody through and into a shower system, you have to tell people, keep them informed of what’s happening […] and then people will be more responsive”; the amount of danger which people perceive themselves to be in, “if something was going to really harm me, I would more or less do anything that I would, to like get it off me” (B3); and greater privacy, “I’d be more willing to do it if there was definite privacy” (A2).

Regarding helping behaviour, several themes emerged from the two control communication conditions. These themes included: desire to help others, “when there’s something out of the ordinary happened, you’re not just thinking of yourself, you’re thinking of the person next to you” (A1), “I’d like to think I’d stay, because you’d think out of public duty you would do” (A2), belief that others will be helpful “[there will probably be] a majority that actually will be able to manage and those people have to help those people who aren’t managing” (B1), “I like to think you know people do what they can, and I’d also think the spirit of human kindness might come into it” (B3), and desire to protect ones family, “if you are with your loved ones and they’re ok, that’s really all you’d think about” (A1), “I would think they need to provide information on your immediate family, how they are, what effect it’s had on them” (A2).

#### Likely behavioural outcomes–respect communication

In both of the respect communication strategy groups, almost all participants stated that they would be willing to comply with the need for decontamination, both in terms of the decontamination shower and decontamination using blue roll. Again, a factor which was suggested to increase compliance was the amount of danger that people perceive themselves to be in, “if it’s life or death you do it” (D1), “If that is what’s needed to do to be like, make sure that I’m safe, then I’d do it” (C3). Similarly, amount of information received was also suggested to increase public compliance, “I want to know first of all what it is, I’m not just going to go and jump in a shower” (C3), “[Without explanation] there’s no real kind of justification, it’s like you want me to do all this stuff, but you don’t know what it is” (D2).

Another theme which emerged from these two conditions was that compliance would be increased to the extent that members of the public trust emergency responders, “you are putting your trust in the emergency services, that they’re looking after you and they’ve got your best interests at heart” (D2).

Themes which emerged around helping behaviour were the same in the two respect communication conditions as in the two control communication conditions, and included: desire to help others, “I probably wouldn’t leg it if I thought I was a danger to society” (C1), “it’s [staying at the scene] not just for your own good, it’s potentially for the good of others too” (C2), “I think I’d help others, most definitely yeah” (D1) ; belief that others will be helpful, “I think if people have got a bit of basic knowledge, until the fire service, the services can get there then we can help each other out a bit” (D3), “I guess you’d have to help each other as well, cos there’s parts you’re not going to be able to reach” (D1); and desire to protect ones family, “you could be passing something on to your family members, so no, I’m not gonna go home” (C2), “I wouldn’t just go home cos I might still be contaminated and pass it on to my family” (D1).

#### Likely psychological outcomes–control communication

A common theme in the control communication strategy groups was a sense of shared fate in response to the emergency, for example, “We’ll look out for each other a little bit here, because we’re a group, to defend ourselves against the unknown and fear” (B1). Some participants highlighted that this sense of shared fate might result in positive outcomes during decontamination, including reduced embarrassment and potentially increased compliance, “I think this whole thing about body and showing our bodies, it goes out the window in moments like that […] all that judgement, all that feeling like people are looking at me, you know, we’re all humans, we’re one, you know, we’ll just have to do it, we just do it” (A3), and desire to help others, “when there’s something out of the ordinary happened, you’re not just thinking of yourself, you’re thinking of the person next to you […] and you want to make sure that you’re all ok” (A1). It was also suggested that emergency responders may be able to foster a sense of shared fate and collective agency (a belief that other members of the group will be supportive in the pursuit of shared group goals, and that group members can work together to challenge and reduce shared stressors: [13], “[someone should say] to the people we’re going to reassure each other, we’re not going to overreact here, you have someone’s going to help you, the person you’ve just made a friend the person’s going to help you” (B1).

#### Likely psychological outcomes–respect communication

A sense of shared fate in response to the emergency was also highlighted by participants in the two respect communication strategy conditions. Comments included: “You’re all kind of in the same…yeah you’re all on the same, literally like in the same environment” (C1), “we’re all in it together” (D1), “everybody’s in the same boat” (D1).

In some groups, participants went further and highlighted some potential positive outcomes arising from a sense of shared fate. These included: reduced embarrassment, “Who cares who’s looking at who really […] Because everyone around there is gonna be in the same scenario” (C1); collective agency and willingness to help others, “I think in that situation there’s like a community spirit so everyone would just be [willing to help others]” (D1); compliance and orderly behaviour, “we’re just waiting like typical British–we’re queueing!” (D1); and promoting compliance through self-policing, “they [people who refuse to comply] don’t want to be near me, I’ll drag em in!” (D1).

### Other themes raised during focus groups

#### Vulnerable groups

One theme which arose across all conditions was that some members of the public would be more vulnerable than others during decontamination. Those who were suggested to be more vulnerable included young children, “I think one of their first priorities would be, like, if there was young children there, babies. I think they would […] maybe that would be the priority” (A3), “so what about the child that doesn’t understand and runs off?” (B3),; those with pre-existing health conditions, “but say you’ve got someone in your family who’s undergoing chemo, and their immune system is so low […] they could be more at risk” (A3), “if someone has mental health issues, that could be exacerbated by erm what happened” (D2); members of religious groups, “I think for religious reasons as well [people might be unwilling to undergo decontamination]” (A3)“there will be people who say well it’s against my religion to get into a communal shower” (C3); people with mobility issues, “some people can’t reach certain parts of their body, so who’s going to help them do that?” (B3); elderly people, “are they going to help people who can’t get their clothes off…you know like an old person?” (D1); and foreign language speakers, “what if people don’t speak English and have people shouting instructions at them?” (D1).

#### Perspective of ‘life and death’ situation

A theme which emerged across all conditions was that the perception of severity of the incident would impact on public willingness to comply with decontamination. Specifically, people would be more willing to comply with decontamination if the incident was perceived to be life-threatening, “if I was in a bad enough state, I think I’d be willing to do anything to save my life” (A3), “if something was going to really harm me, I would more or less do anything that I would, to like get it off me, even if that involved stripping in front of a load of men” (B3), “Like if it was a matter of life or death–you just do it” (C1).

#### Public education

A subject that was mentioned in some of the groups was that, having taken part in these focus groups, participants felt that some form of pre-incident public education about what to do during decontamination would be beneficial. Comments included: “there needs to be more general public information about what happens in this situations” (B1), “It probably be difficult not to scaremonger people but if there was something where there was some information, that, which is obviously very simple, if this if this was to happen do a, b, c you know like on the adverts with the stroke? You know, and if I think if people have got a bit of basic knowledge, until the fire service, the services can get there then we can help each other out a bit maybe a bit more” (D3).

### Questionnaire results

#### Between groups differences

Results from a series of two-way ANOVAs revealed various significant differences between groups. There was no significant interaction between the effects of level of communication and decontamination condition on level of embarrassment in undergoing a decontamination shower (*F* (1, 58) = .31, *n*.*s*.). There was a significant main effect of communication condition on level of embarrassment in undergoing a decontamination shower (*F* (1, 58) = 6.94, *p* < .05), with those in the respect communication condition reporting significantly less embarrassment in undergoing a decontamination shower (*M* = 5.58) than those in the control communication condition (*M* = 4.23). There was no significant main effect of decontamination condition on level of embarrassment in undergoing a decontamination shower (*F* (1, 58) = .03, *n*.*s*.).

There was no significant interaction between communication condition and decontamination condition on perceived ease of undergoing a decontamination shower (*F* (1, 58) = .90, *n*.*s*.). There was a significant main effect of decontamination condition on perceived ease of undergoing a decontamination shower (*F* (1, 58) = 5.91, *p* < .05), with those in the wet decontamination only condition reporting significantly greater perceived ease of undergoing a decontamination shower (*M* = 6.03) than those in the dry decontamination and wet decontamination condition (*M* = 5.22). There was no significant main effect of communication condition on perceived ease of undergoing a decontamination shower (*F* (1, 58) = .12, *n*.*s*.).

There was no significant interaction between communication condition and decontamination condition on perceived effectiveness of a decontamination shower (*F* (1, 58) = .30, *n*.*s*.). There was a significant main effect of decontamination condition on perceived effectiveness of a decontamination shower (*F* (1, 58) = 4.28, *p* < .05), with those in the wet decontamination only condition reporting significantly greater perceived effectiveness of a decontamination shower (*M* = 5.63) than those in the dry decontamination and wet decontamination condition (*M* = 4.97). There was no significant main effect of communication condition on perceived ease of undergoing a decontamination shower (*F* (1, 58) = 2.22, *n*.*s*.).

There was no significant interaction between communication condition and decontamination condition on likelihood to seek further treatment following decontamination (*F* (1, 58) = .11, *n*.*s*.). There was a significant main effect of communication condition on likelihood to seek further treatment following decontamination (*F* (1, 58) = 3.91, *p* = .05), with those in the respect communication condition reporting significantly lower likelihood to seek further treatment following decontamination (*M* = 6.25) than those in the control communication condition (*M* = 6.70). There was no significant main effect of decontamination condition on likelihood to seek further treatment following decontamination (*F* (1, 58) = .38, *n*.*s*.).

The remaining two-way ANOVAs revealed no other significant differences between groups at time 2. See Table 2 for the M and SD from the two-way ANOVAs, and Table 3 for the F and P values from two-way ANOVAs.

**Table 2:** *M* and *SD* from two-way ANOVAs.

		Decontamination				Communication			
		Wet only		Dry & wet		Control		Respect	
Variable	*N*	*M*	*SD*	*M*	*SD*	*M*	*SD*	*M*	*SD*
**Knowledge & confidence**	63	4.20	1.54	4.27	1.41	4.09	1.58	4.38	1.35
**Responder actions**	63	5.66	1.11	5.91	.90	5.81	1.01	5.77	1.02
**Social support**	62	4.82	1.25	4.90	1.19	4.69	1.26	5.03	1.15
**Comfortable**	62	6.17	1.37	6.09	1.09	6.16	1.29	6.10	1.16
**Embarrassed**	62	4.90	2.26	4.91	1.99	4.23	2.25	5.59	1.75
**Easy**	62	6.03	1.19	5.22	1.41	5.58	1.43	5.65	1.31
**Effective**	62	5.63	1.25	4.97	1.40	5.06	1.50	5.52	1.18
**Willing**	62	6.57	.73	6.22	1.36	6.30	1.26	6.47	.95
**Anxious**	62	6.03	1.25	6.31	1.03	6.37	.72	6.00	1.41
**Further treatment**	62	6.40	.97	6.53	.88	6.70	.70	6.25	1.05

**Table 3:** *F* values and *p* values from two-way ANOVAs.

	Decontamination		Communication		Interaction	
Variable	*F*	*p*	*F*	*p*	*F*	*P*
**Knowledge & confidence**	.03	.86	.67	.42	2.97	.09
**Responder actions**	.91	.34	.04	.84	.06	.80
**Social support**	.03	.87	1.30	.26	1.21	.28
**Comfortable**	.05	.82	.02	.88	1.65	.20
**Embarrassed**	.03	.87	6.94	.01*	.31	.58
**Easy**	5.91	.02*	.12	.73	.02	.90
**Effective**	4.28	.04*	2.22	.14	.30	.59
**Willing**	1.58	.21	.39	.54	.15	.70
**Anxious**	.96	.33	1.81	.18	1.46	.23
**Further treatment**	.38	.54	3.91	.05*	.11	.74

*Significant at .05 level

Results from a series of one-way ANOVAs revealed some significant differences between groups. Those in the respect communication condition were significantly more likely to comply with the use of blue roll for decontamination (*M* = 6.47) than those in the control communication condition (*M* = 5.20) (*F* (1, 30) = 6.16, *p* < .05). Those in the respect communication condition reported significantly less embarrassment in using blue roll for decontamination (*M* = 6.35) compared to those in the control communication condition (M = 4.47) (*F* (1, 30) = 13.81, *p* = .001). Those in the respect communication condition reported significantly greater perceived ease of using blue roll for decontamination (*M* = 5.65) than those in the control communication condition (*M* = 4.60) (*F* (1, 30) = 4.13, *p* = .05).The one-way ANOVAs revealed no other significant differences between groups at Time 2.

#### Within groups differences

The response rate at Time 3 was fairly low (24%); however, this response rate was large enough to enable us to carry out a one-way within-subjects ANOVA in order to compare knowledge and confidence in taking protective actions between Time 1, Time 2, and Time 3. There was a significant effect of time on individuals’ perceived knowledge and of, and confidence in, protecting themselves and others in the event of a chemical incident, Wilks’ Lambda = .49, *F* (2, 13) = 6.73, *p* < .05. This was due to a significant increase in perceived knowledge and confidence from Time 1 (*M* = 2.82) to Time 2 (*M* = 4.27) (*p* < .05) and a significant increase in perceived knowledge and confidence from Time 1 to Time 3 (*M* = 4.48) (*p* < .05).

## Discussion

Findings from this study provide an initial insight into how members of the public perceive Initial Operational Response dry decontamination methods, in comparison to existing methods of wet decontamination. Whilst there are some limitations to our approach which we have summarised in the Limitations section below, our findings also provide an understanding of how different responder management strategies might affect public perceptions of decontamination methods. Overall, the decontamination shower was perceived to be more effective than blue roll among participants in the two conditions in which both interventions were received. However, perceptions about the use of blue roll were more positive in the condition in which participants received more information about the efficacy of using blue roll. Questionnaire results supported this, showing that those in the respect management condition would be significantly more willing to comply with use of blue roll than those in the control management condition. This is as would be expected based on the findings from previous research [11], and suggests that providing detailed information about how and why blue roll should be used may increase the perceived acceptability of using blue roll as part of the decontamination process, and may therefore increase willingness to comply with the use of blue roll.

Findings regarding public perceptions of the acceptability of using blue roll have important implications for new IOR procedures in which dry decontamination would be used [6]. Based on current findings, in order to ensure that blue roll is perceived as an acceptable intervention, detailed information must be provided about why dry decontamination is necessary, and how decontamination using absorbent materials should be undertaken. A similar communication strategy has been found to be effective in promoting compliance with undergoing a decontamination shower [4].

Almost all participants stated that they would want to receive further treatment following decontamination, as would be expected based on previous research [11]. Expressions of desire to seek further treatment did not appear to differ as a consequence of the type of decontamination intervention or amount of information provided, when examining focus group transcripts. However, questionnaire data revealed that those in the respect management strategy conditions were significantly less likely to report a desire to seek further treatment than those in the control management strategy conditions. The desire for further treatment following decontamination requires investigation, as it could result in large numbers of people attending, and potentially overwhelming, healthcare facilities during mass casualty incidents. The reduced desire to seek further treatment in the respect management strategy condition suggests that the provision of effective communication may make people less likely to seek further treatment. Several participants highlighted that they would like to receive further information following decontamination, in the form of an information leaflet or helpline, and it is possible that provision of this type of follow up information could reduce public desire to seek further treatment following decontamination. It is not currently standard practice to provide follow up information after decontamination, but findings from this study suggest that development of such information could provide reassurance to those affected, and reduce the likelihood that they would seek further treatment following decontamination.

As expected, perceptions of the amount of information provided were more negative among participants who received the control communication strategy than among those who received the respect communication strategy. However, many of those in the respect communication strategy still felt that the level of information they had been provided with would not be sufficient during a real incident. This highlights how much information people are likely to need during decontamination. Those in the respect communication conditions were more likely to report that they would be willing to comply with the need for decontamination, which is in line with findings from previous research [3–5], and emphasises the importance of providing effective communication during this type of incident.

During the focus groups, several participants suggested that providing pre-incident public education may help to prepare people to take protective actions during an incident involving decontamination. Results from the questionnaire data revealed that participants in all conditions reported increased knowledge and confidence about taking protective actions during decontamination after taking part in the focus groups. Interestingly, this increased knowledge and confidence was maintained at a 3 month follow up. The idea of providing members of the public with pre-incident information about what to do during decontamination could be beneficial, as it could allow vital actions (such as evacuation and disrobe) to be undertaken prior to the arrival of the emergency services. This study provides preliminary support for the idea that it may be possible to educate members of the public about appropriate actions to take, prior to an incident involving decontamination occurring. Although there is limited evidence relating to the effectiveness of pre-incident information campaigns, the current finding is in-line with the findings from a recent literature review which examined the effectiveness of pre-incident public education for natural disasters and acts of terrorism [14]. However, as noted above, this study examined perceived knowledge and confidence in relation to taking recommended decontamination actions, and did not test participants’ actual knowledge. The possibility of developing pre-incident public education for incidents involving decontamination would benefit from further research, for example a trial comparing the effectiveness of different pre-incident public education campaigns for increasing public knowledge about protective actions to take during incidents requiring decontamination.

### Limitations

A limitation of the current research is that participants were asked to visualise that they were involved in an incident requiring decontamination, without actually having to undergo the decontamination process. Participants may therefore have found it difficult to accurately visualise the type of incident described in the scenario, and therefore to accurately imagine how they would act during this type of situation, or what their needs might be. A second limitation of the current research is that, while all participants completed the pre-focus group and post-focus group questionnaires, the number of participants who returned the 3 month questionnaire was fairly low 15 (24%) and so the representativeness of the sample may be questioned. Further, participants’ were asked about their perceived knowledge and confidence in taking protective decontamination actions. Whilst it is encouraging that participants’ perceived knowledge and confidence increased after taking part in the focus group, and was maintained 3 months later, it should be noted that participants’ actual knowledge about appropriate protective actions to take was not measured.

Overall, findings from this study illustrate the importance of understanding public perceptions of different decontamination interventions. Certain decontamination interventions (e.g. a decontamination shower) are perceived to be more effective than others (e.g. dry decontamination), and this perception could play a key role in public willingness to comply with recommended decontamination interventions. Crucially, employing a management strategy that includes effective communication about why decontamination is necessary and what the decontamination process will involve could improve public compliance, and reduce public desire to seek further treatment following decontamination.

## Supporting information

S1 Text Focus group scenario. (DOCX)

S2 Text Focus group interventions. (DOCX)

S3 Text Pre-focus group questionnaire. (DOCX)

S4 Text Post-focus group questionnaire. (DOCX)

S5 Text Three month follow up questionnaire. (DOCX)

S6 Text Focus group discussion guide. (DOCX)

S7 Text Information sheet. (DOCX)

S8 Text Consent form. (DOC)
