# Supplementary material for: Public perceptions of emergency decontamination: Effects of intervention type and responder management strategy during a focus group study
Source: PLoS One. 2018 Apr 13;13(4):e0195922. doi: 10.1371/journal.pone.0195922 (PMC5898741; doi:10.1371/journal.pone.0195922)
Supplement: S1 Text — (DOCX) [file pone.0195922.s001.docx]

**S1 Text: Focus group scenario**

You are at a train station, waiting for a train. It is just after 5pm on a Tuesday afternoon, and the train station is busy with commuters. You notice that near to where you are standing, a man is watering some large potted plants with a backpack type sprayer. You notice a strong smell, a bit like rotten eggs. Without warning, the man turns from the plants which he is watering, and begins to run through the crowd of people, spraying them with the liquid in his backpack sprayer. Some of the liquid lands on your skin. Shortly after this, you notice that people near to you are coughing. You begin to cough too, and your vision becomes blurry. You realise that your symptoms are probably caused by the liquid which is on your skin.
