# Supplementary material for: Public perceptions of emergency decontamination: Effects of intervention type and responder management strategy during a focus group study
Source: PLoS One. 2018 Apr 13;13(4):e0195922. doi: 10.1371/journal.pone.0195922 (PMC5898741; doi:10.1371/journal.pone.0195922)
Supplement: S2 Text — (DOCX) [file pone.0195922.s002.docx]

**S2 Text: Focus group interventions**

*Condition 1*

After a few minutes, a small team of emergency responders arrive. They tell you, and others nearby, to follow them to a location outside the train station. They then ask you to remove the top layer of your clothes, down to your underwear. Emergency responders set up a large shower system, using hoses from their fire engines. Emergency responders tell you and the others affected to form a queue and wait to enter the large shower system. The shower has two separate entrances, with women entering on one side, and men on the other. You are told to remove your underwear, and then enter the shower one by one. You walk through the shower. After going through the shower, emergency responders give you a towel to dry yourself. You are then provided with some temporary clothing to wear, and are told by emergency responders that you are now free to leave the scene and return home.

*Condition 2*

After a few minutes, a small team of emergency responders arrive. They tell you, and others nearby, to follow them to a location outside the train station. They then ask you to remove the top layer of your clothes, down to your underwear. They give you some blue roll, and tell you to use it to wipe the liquid off your skin. While you are wiping the liquid off your skin, emergency responders set up a large shower system, using hoses from their fire engines. Emergency responders tell you and the others affected to form a queue and wait to enter the large shower system. The shower has two separate entrances, with women entering on one side, and men on the other. You are told to remove your underwear, and then enter the shower one by one. You walk through the shower. After going through the shower, emergency responders give you a towel to dry yourself. You are then provided with some temporary clothing to wear, and are told by emergency responders that you are now free to leave the scene and return home.

*Condition 3*

After a few minutes, a small team of emergency responders arrive. They explain that you have potentially come into contact with a chemical, and to prevent further contamination, they ask you to follow them to a location outside the train station. They then ask you to remove the top layer of your clothes, down to your underwear. They explain that removing the top layer of your clothes will remove 80-90% of any contaminant which you may have come into contact with, and will therefore help to protect you. Emergency responders set up a large shower system, using hoses from their fire engines. Emergency responders explain that going through the shower will remove any contaminant which may remain on your skin. Emergency responders ask you and the others affected to form a queue and wait to enter the large shower system. The shower has two separate entrances, with women entering on one side, and men on the other. You are asked to remove your underwear, as this will help to ensure that all remaining contaminant is removed from your skin during the shower. You are asked to enter the shower one by one, raising your arms, and turning 90 degrees every few seconds. You walk through the shower. After going through the shower, emergency responders give you a towel to dry yourself, and you are provided with some temporary clothing to wear. Emergency responders explain that having taken your clothes off, wiped yourself with blue roll, and gone through the shower, you are now clean. They provide you with further information on what to do if you experience any further symptoms, and then advise you that you are free to leave the scene.

*Condition 4*

After a few minutes, a small team of emergency responders arrive. They explain that you have potentially come into contact with a chemical, and to prevent further contamination, they ask you to follow them to a location outside the train station. They then ask you to remove the top layer of your clothes, down to your underwear. They explain that removing the top layer of your clothes will remove 80-90% of any contaminant which you may have come into contact with, and will therefore help to protect you. They give you some blue roll, and ask you to use it to wipe the liquid off your skin. They explain that using the blue roll to wipe yourself down will remove any contaminant which may remain on your skin, and will therefore help to reduce any risks from the contaminant. While you are wiping the liquid off your skin, emergency responders set up a large shower system, using hoses from their fire engines. Emergency responders explain that going through the shower will remove any contaminant which may remain on your skin. Emergency responders ask you and the others affected to form a queue and wait to enter the large shower system. The shower has two separate entrances, with women entering on one side, and men on the other. You are asked to remove your underwear, as this will help to ensure that all remaining contaminant is removed from your skin during the shower. You are asked to enter the shower one by one, raising your arms, and turning 90 degrees every few seconds. You walk through the shower. After going through the shower, emergency responders give you a towel to dry yourself, and you are provided with some temporary clothing to wear. Emergency responders explain that having taken your clothes off, wiped yourself with blue roll, and gone through the shower, you are now clean. They provide you with further information on what to do if you experience any further symptoms, and then advise you that you are free to leave the scene.
