# Supplementary material for: Public perceptions of emergency decontamination: Effects of intervention type and responder management strategy during a focus group study
Source: PLoS One. 2018 Apr 13;13(4):e0195922. doi: 10.1371/journal.pone.0195922 (PMC5898741; doi:10.1371/journal.pone.0195922)
Supplement: S4 Text — (DOCX) [file pone.0195922.s004.docx]

**S4 Text: Post-focus group questionnaire**

**1. If a real incident of this type were to occur, I would know what actions to take to protect myself.**

| Strongly disagree | 1 | 2 | 3 | 4 | 5 | 6 | 7 | Strongly agree |
| --- | --- | --- | --- | --- | --- | --- | --- | --- |

**2. If a real incident of this type were to occur, I would know what actions to take to protect my loved ones.**

| Strongly disagree | 1 | 2 | 3 | 4 | 5 | 6 | 7 | Strongly agree |
| --- | --- | --- | --- | --- | --- | --- | --- | --- |

**3. If a real incident of this type were to occur, I would feel confident that I could successfully undertake appropriate actions in order to protect myself.**

| Strongly disagree | 1 | 2 | 3 | 4 | 5 | 6 | 7 | Strongly agree |
| --- | --- | --- | --- | --- | --- | --- | --- | --- |

**4. If a real incident of this type were to occur, I would feel confident that I could successfully undertake appropriate actions in order to protect my loved ones.**

| Strongly disagree | 1 | 2 | 3 | 4 | 5 | 6 | 7 | Strongly agree |
| --- | --- | --- | --- | --- | --- | --- | --- | --- |

**5. Please describe any actions which you would take if an incident of this type were to occur.**

|  |
| --- |

**6. I think that the emergency services would behave in a respectful way when managing this type of incident.**

| Strongly disagree | 1 | 2 | 3 | 4 | 5 | 6 | 7 | Strongly agree |
| --- | --- | --- | --- | --- | --- | --- | --- | --- |

**7. I think that the emergency services would behave in a fair way when managing this type of incident.**

| Strongly disagree | 1 | 2 | 3 | 4 | 5 | 6 | 7 | Strongly agree |
| --- | --- | --- | --- | --- | --- | --- | --- | --- |

**8. I think that the emergency services would behave in a forceful way when managing this type of incident.**

| Strongly disagree | 1 | 2 | 3 | 4 | 5 | 6 | 7 | Strongly agree |
| --- | --- | --- | --- | --- | --- | --- | --- | --- |

**9. If this was a real incident, I would expect emotional support from other members of the public who were involved.**

| Strongly disagree | 1 | 2 | 3 | 4 | 5 | 6 | 7 | Strongly agree |
| --- | --- | --- | --- | --- | --- | --- | --- | --- |

**10. If this was a real incident, I would expect to receive help from other members of the public who were involved.**

| Strongly disagree | 1 | 2 | 3 | 4 | 5 | 6 | 7 | Strongly agree |
| --- | --- | --- | --- | --- | --- | --- | --- | --- |

**11. If this was a real incident, I would be willing to help other members of the public.**

| Strongly disagree | 1 | 2 | 3 | 4 | 5 | 6 | 7 | Strongly agree |
| --- | --- | --- | --- | --- | --- | --- | --- | --- |

*Question for all conditions*

**12. If this were a real incident I would feel comfortable undergoing a decontamination shower in order to remove a contaminant from my skin.**

| Strongly disagree | 1 | 2 | 3 | 4 | 5 | 6 | 7 | Strongly agree |
| --- | --- | --- | --- | --- | --- | --- | --- | --- |

*Extra question for condition with dry decontamination*

**12a. If this were a real incident I would feel comfortable using the blue roll to remove a contaminant from my skin.**

| Strongly disagree | 1 | 2 | 3 | 4 | 5 | 6 | 7 | Strongly agree |
| --- | --- | --- | --- | --- | --- | --- | --- | --- |

*Question for all conditions*

**13. If this were a real incident, I would feel embarrassed undergoing a decontamination shower in order to remove a contaminant from my skin.**

| Strongly disagree | 1 | 2 | 3 | 4 | 5 | 6 | 7 | Strongly agree |
| --- | --- | --- | --- | --- | --- | --- | --- | --- |

*Extra question for condition with dry decontamination*

**13a. If this were a real incident, I would feel embarrassed using the blue roll to remove a contaminant from my skin.**

| Strongly disagree | 1 | 2 | 3 | 4 | 5 | 6 | 7 | Strongly agree |
| --- | --- | --- | --- | --- | --- | --- | --- | --- |

*Question for all conditions*

**14. If this were a real incident, I think I would find it easy to undergo a decontamination shower in order to remove a contaminant from my skin.**

| Strongly disagree | 1 | 2 | 3 | 4 | 5 | 6 | 7 | Strongly agree |
| --- | --- | --- | --- | --- | --- | --- | --- | --- |

*Extra question for condition with dry decontamination*

**14a. If this were a real incident, I think I would find it easy to use blue roll to remove a contaminant from my skin.**

| Strongly disagree | 1 | 2 | 3 | 4 | 5 | 6 | 7 | Strongly agree |
| --- | --- | --- | --- | --- | --- | --- | --- | --- |

*Question for all conditions*

**15. If this were a real incident, I think that undergoing a decontamination shower would be an effective way to remove a contaminant from my skin.**

| Strongly disagree | 1 | 2 | 3 | 4 | 5 | 6 | 7 | Strongly agree |
| --- | --- | --- | --- | --- | --- | --- | --- | --- |

*Question for condition with dry decontamination*

**15a. If this were a real incident, I think that using blue roll would be an effective way to remove a contaminant from my skin.**

| Strongly disagree | 1 | 2 | 3 | 4 | 5 | 6 | 7 | Strongly agree |
| --- | --- | --- | --- | --- | --- | --- | --- | --- |

*Question for all conditions*

**16. I would be willing to undergo a decontamination shower during a real life incident of this kind.**

| Strongly disagree | 1 | 2 | 3 | 4 | 5 | 6 | 7 | Strongly agree |
| --- | --- | --- | --- | --- | --- | --- | --- | --- |

*Question for condition with dry decontamination*

**16a. I would be willing to use blue roll to decontaminate myself during a real life incident of this kind.**

| Strongly disagree | 1 | 2 | 3 | 4 | 5 | 6 | 7 | Strongly agree |
| --- | --- | --- | --- | --- | --- | --- | --- | --- |

**17. If a real incident of this kind occurred, I would feel anxious.**

| Strongly disagree | 1 | 2 | 3 | 4 | 5 | 6 | 7 | Strongly agree |
| --- | --- | --- | --- | --- | --- | --- | --- | --- |

**18. If this were a real incident, I would feel the need to seek further treatment after [using the blue roll to remove the contaminant from my skin/ using the blue roll to remove the contaminant from my skin, and then undergoing a decontamination shower].**

| Strongly disagree | 1 | 2 | 3 | 4 | 5 | 6 | 7 | Strongly agree |
| --- | --- | --- | --- | --- | --- | --- | --- | --- |
