# Supplementary material for: Public perceptions of emergency decontamination: Effects of intervention type and responder management strategy during a focus group study
Source: PLoS One. 2018 Apr 13;13(4):e0195922. doi: 10.1371/journal.pone.0195922 (PMC5898741; doi:10.1371/journal.pone.0195922)
Supplement: S5 Text — (DOCX) [file pone.0195922.s005.docx]

**S5 Text: Three month follow up questionnaire**

**1. If a real incident of this type were to occur, I would know what actions to take to protect myself.**

| *Strongly disagree* | 1 | 2 | 3 | 4 | 5 | 6 | 7 | *Strongly agree* |
| --- | --- | --- | --- | --- | --- | --- | --- | --- |

**2. If a real incident of this type were to occur, I would know what actions to take to protect my loved ones.**

| *Strongly disagree* | 1 | 2 | 3 | 4 | 5 | 6 | 7 | *Strongly agree* |
| --- | --- | --- | --- | --- | --- | --- | --- | --- |

**3. If a real incident of this type were to occur, I would feel confident that I could successfully undertake appropriate actions in order to protect myself.**

| *Strongly disagree* | 1 | 2 | 3 | 4 | 5 | 6 | 7 | *Strongly agree* |
| --- | --- | --- | --- | --- | --- | --- | --- | --- |

**4. If a real incident of this type were to occur, I would feel confident that I could successfully undertake appropriate actions in order to protect my loved ones.**

| *Strongly disagree* | 1 | 2 | 3 | 4 | 5 | 6 | 7 | *Strongly agree* |
| --- | --- | --- | --- | --- | --- | --- | --- | --- |

**5. Please describe any actions which you would take if an incident of this type were to occur.**

|  |
| --- |
