# Supplementary material for: Public perceptions of emergency decontamination: Effects of intervention type and responder management strategy during a focus group study
Source: PLoS One. 2018 Apr 13;13(4):e0195922. doi: 10.1371/journal.pone.0195922 (PMC5898741; doi:10.1371/journal.pone.0195922)
Supplement: S6 Text — (DOCX) [file pone.0195922.s006.docx]

**S6 Text: Focus group discussion guide**

*Condition 1*

**1. Ask participants to complete consent form and database consent form.**

**2. Explain the nature of the study:**

Thank you for agreeing to take part in this focus group. Before we start, I’d just like to remind you that all information that you give will be confidential, and any published data from these focus groups will be anonymous. I’d also like to remind you that we are recording these focus groups with digital recorders and video cameras – these will only be used to allow us to analyse the data collected, and will only be shared with members of the immediate research team.

In this study, we are interested to find out about people’s perceptions of decontamination. Decontamination is an intervention which would be used in the event of a chemical release in order to remove a chemical from someone’s skin. There are various different methods of decontamination, and this study is specifically designed to understand what people think about these different decontamination methods. So to understand more about what decontamination looks like, please have a look at this picture.

**3. Provide participants with the scenario and ask them to read it.**

**4. Provide participants with the pre-focus group questionnaire and ask them to complete it.**

**5. Ask the following questions, and facilitate discussion of these:**

1. How do you think you would feel if an incident of this type were to occur?

2. What would your main concerns be?

3. What would you do if you found yourself in this scenario?

4. What actions do you think emergency responders would take during an incident of this type?

**6. Ask participants to read Intervention for Condition 1.**

**7. Ask participants the following questions, and facilitate discussion of these:**

5. Would you feel confident that emergency responders could manage the incident effectively?

5a. If so/ not, why?

6. How do you think emergency responders would behave during an incident of this type?

6a. Would they treat you fairly/ with respect? If so/ not, why?

7. If an incident of this type were to occur, would you be willing to remain at the scene and undergo a decontamination shower?

8. Would you want to seek further treatment after undergoing a decontamination shower?

8a. If so / not, why?

9. If a real life incident of this type occurred, would you be willing to undergo a decontamination shower?

10. If this were a real incident, would you feel comfortable undergoing a decontamination shower?

11. If this were a real incident, do you think you would find it easy to undergo a decontamination shower?

12. Would you feel confident that you were clean after undergoing a decontamination shower?

13. Do you think the level of information provided in this scenario would be sufficient?

13a. If not, why?

14. What information do you think you would need in this scenario?

15. What information do you think you would need in order to successfully undergo a decontamination shower?

16. Would you be willing to remove your clothes in order to undergo a decontamination shower?

16a. If not, why?

17. Do you think effort would be made to protect your privacy during decontamination?

17a. If not, why?

**8. Ask participants to complete the post-focus group questionnaire.**

**9. Give participants the debrief statement, £60 of gift vouchers, and ask them to sign the Record of Reimbursement to show that they have received these. Remind participants that we will contact them in 3 months time to ask them to complete a short follow-up questionnaire.**

*Condition 2*

**1. Ask participants to complete consent form and database consent form.**

**2. Explain the nature of the study:**

Thank you for agreeing to take part in this focus group. Before we start, I’d just like to remind you that all information that you give will be confidential, and any published data from these focus groups will be anonymous. I’d also like to remind you that we are recording these focus groups with digital recorders and video cameras – these will only be used to allow us to analyse the data collected, and will only be shared with members of the immediate research team.

In this study, we are interested to find out about people’s perceptions of decontamination. Decontamination is an intervention which would be used in the event of a chemical release in order to remove a chemical from someone’s skin. There are various different methods of decontamination, and this study is specifically designed to understand what people think about these different decontamination methods. So to understand more about what decontamination looks like, please have a look at this picture.

**3. Provide participants with the scenario and ask them to read it.**

**4. Provide participants with the pre-focus group questionnaire and ask them to complete it.**

**5. Ask the following questions, and facilitate discussion of these:**

1. How do you think you would feel if an incident of this type were to occur?

2. What would your main concerns be?

3. What would you do if you found yourself in this scenario?

4. What actions do you think emergency responders would take during an incident of this type?

**6. Ask participants to read Intervention for Condition 2.**

**7. Ask participants the following questions, and facilitate discussion of these:**

5. Would you feel confident that emergency responders could manage the incident effectively?

5a. If so/ not, why?

6. How do you think emergency responders would behave during an incident of this type?

6a. Would they treat you fairly/ with respect? If so/ not, why?

7. If an incident of this type were to occur, would you be willing to remain at the scene and:

decontaminate yourself using blue roll?

undergo a decontamination shower?

8. Would you want to seek further treatment after:

wiping yourself down with blue roll?

undergoing a decontamination shower?

8a. If so / not, why?

9. If a real life incident of this type occurred, would you be willing to:

decontaminate yourself using blue roll?

undergo a decontamination shower?

10. If this were a real incident, would you feel comfortable:

using blue roll to decontaminate yourself?

undergoing a decontamination shower?

11. If this were a real incident, do you think you would find it easy to:

decontaminate yourself using blue roll?

undergo a decontamination shower?

12. Would you feel confident that you were clean after:

wiping yourself down with blue roll?

undergoing a decontamination shower?

13. Do you think the level of information provided in this scenario would be sufficient?

13a. If not, why?

14. What information do you think you would need in this scenario?

15. What information do you think you would need in order to successfully:

decontaminate yourself using blue roll?

undergo a decontamination shower?

16. Would you be willing to remove your clothes in order to:

wipe yourself down with blue roll?

undergo a decontamination shower?

16a. If not, why?

17. Do you think effort would be made to protect your privacy during decontamination?

17a. If not, why?

**8. Ask participants to complete the post-focus group questionnaire.**

**9. Give participants the debrief statement, £60 of gift vouchers, and ask them to sign the Record of Reimbursement to show that they have received these. Remind participants that we will contact them in 3 months time to ask them to complete a short follow-up questionnaire.**

*Condition 3*

**1. Ask participants to complete consent form and database consent form.**

**2. Explain the nature of the study:**

Thank you for agreeing to take part in this focus group. Before we start, I’d just like to remind you that all information that you give will be confidential, and any published data from these focus groups will be anonymous. I’d also like to remind you that we are recording these focus groups with digital recorders and video cameras – these will only be used to allow us to analyse the data collected, and will only be shared with members of the immediate research team.

In this study, we are interested to find out about people’s perceptions of decontamination. Decontamination is an intervention which would be used in the event of a chemical release in order to remove a chemical from someone’s skin. There are various different methods of decontamination, and this study is specifically designed to understand what people think about these different decontamination methods. So to understand more about what decontamination looks like, please have a look at this picture.

**3. Provide participants with the scenario and ask them to read it.**

**4. Provide participants with the pre-focus group questionnaire and ask them to complete it.**

**5. Ask the following questions, and facilitate discussion of these:**

1. How do you think you would feel if an incident of this type were to occur?

2. What would your main concerns be?

3. What would you do if you found yourself in this scenario?

4. What actions do you think emergency responders would take during an incident of this type?

**6. Ask participants to read Intervention for Condition 3.**

**7. Ask participants the following questions, and facilitate discussion of these:**

5. Would you feel confident that emergency responders could manage the incident effectively?

5a. If so/ not, why?

6. How do you think emergency responders would behave during an incident of this type?

6a. Would they treat you fairly/ with respect? If so/ not, why?

7. If an incident of this type were to occur, would you be willing to remain at the scene and undergo a decontamination shower?

8. Would you want to seek further treatment after undergoing a decontamination shower?

8a. If so / not, why?

9. If a real life incident of this type occurred, would you be willing to undergo a decontamination shower?

10. If this were a real incident, would you feel comfortable undergoing a decontamination shower?

11. If this were a real incident, do you think you would find it easy to undergo a decontamination shower?

12. Would you feel confident that you were clean after undergoing a decontamination shower?

13. Do you think the level of information provided in this scenario would be sufficient?

13a. If not, why?

14. What information do you think you would need in this scenario?

15. What information do you think you would need in order to successfully undergo a decontamination shower?

16. Would you be willing to remove your clothes in order to undergo a decontamination shower?

16a. If not, why?

17. Do you think effort would be made to protect your privacy during decontamination?

17a. If not, why?

**8. Ask participants to complete the post-focus group questionnaire.**

**9. Give participants the debrief statement, £60 of gift vouchers, and ask them to sign the Record of Reimbursement to show that they have received these. Remind participants that we will contact them in 3 months time to ask them to complete a short follow-up questionnaire.**

*Condition 4*

**1. Ask participants to complete consent form and database consent form.**

**2. Explain the nature of the study:**

Thank you for agreeing to take part in this focus group. Before we start, I’d just like to remind you that all information that you give will be confidential, and any published data from these focus groups will be anonymous. I’d also like to remind you that we are recording these focus groups with digital recorders and video cameras – these will only be used to allow us to analyse the data collected, and will only be shared with members of the immediate research team.

In this study, we are interested to find out about people’s perceptions of decontamination. Decontamination is an intervention which would be used in the event of a chemical release in order to remove a chemical from someone’s skin. There are various different methods of decontamination, and this study is specifically designed to understand what people think about these different decontamination methods. So to understand more about what decontamination looks like, please have a look at this picture.

**3. Provide participants with the scenario and ask them to read it.**

**4. Provide participants with the pre-focus group questionnaire and ask them to complete it.**

**5. Ask the following questions, and facilitate discussion of these:**

1. How do you think you would feel if an incident of this type were to occur?

2. What would your main concerns be?

3. What would you do if you found yourself in this scenario?

4. What actions do you think emergency responders would take during an incident of this type?

**6. Ask participants to read Intervention for Condition 4.**

**7. Ask participants the following questions, and facilitate discussion of these:**

5. Would you feel confident that emergency responders could manage the incident effectively?

5a. If so/ not, why?

6. How do you think emergency responders would behave during an incident of this type?

6a. Would they treat you fairly/ with respect? If so/ not, why?

7. If an incident of this type were to occur, would you be willing to remain at the scene and:

decontaminate yourself using blue roll?

undergo a decontamination shower?

8. Would you want to seek further treatment after:

wiping yourself down with blue roll?

undergoing a decontamination shower?

8a. If so / not, why?

9. If a real life incident of this type occurred, would you be willing to:

decontaminate yourself using blue roll?

undergo a decontamination shower?

10. If this were a real incident, would you feel comfortable:

using blue roll to decontaminate yourself?

undergoing a decontamination shower?

11. If this were a real incident, do you think you would find it easy to:

decontaminate yourself using blue roll?

undergo a decontamination shower?

12. Would you feel confident that you were clean after:

wiping yourself down with blue roll?

undergoing a decontamination shower?

13. Do you think the level of information provided in this scenario would be sufficient?

13a. If not, why?

14. What information do you think you would need in this scenario?

15. What information do you think you would need in order to successfully:

decontaminate yourself using blue roll?

undergo a decontamination shower?

16. Would you be willing to remove your clothes in order to:

wipe yourself down with blue roll?

undergo a decontamination shower?

16a. If not, why?

17. Do you think effort would be made to protect your privacy during decontamination?

17a. If not, why?

**8. Ask participants to complete the post-focus group questionnaire.**

**9. Give participants the debrief statement, £60 of gift vouchers, and ask them to sign the Record of Reimbursement to show that they have received these. Remind participants that we will contact them in 3 months time to ask them to complete a short follow-up questionnaire.**
