# Supplementary material for: Public perceptions of emergency decontamination: Effects of intervention type and responder management strategy during a focus group study
Source: PLoS One. 2018 Apr 13;13(4):e0195922. doi: 10.1371/journal.pone.0195922 (PMC5898741; doi:10.1371/journal.pone.0195922)
Supplement: S7 Text — (DOCX) [file pone.0195922.s007.docx]

[
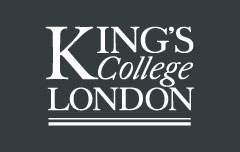
](http://www.google.co.uk/url?sa=i&rct=j&q=&esrc=s&frm=1&source=images&cd=&cad=rja&uact=8&ved=0CAcQjRxqFQoTCLPW5si-v8gCFYpAFAod5hAJpw&url=http://www.kcl.ac.uk/prospectus/graduate/master-of-laws&psig=AFQjCNEQClsKei63zmw18YTkEvJmj5OTjw&ust=1444826887670530)

September 2015

**S7 Text**

**INFORMATION SHEET**

**Focus group on emergency response**

Thank you very much for considering being a volunteer in this research. Before you decide to take part, it is important that you understand why it is being carried out and what it will involve. Please take the time to read the following information carefully, and contact us if there is anything that is unclear, or if you would like more information.

**What is the purpose of the study?**

In the event of an incident where a chemical is released, such as a factory accident or a deliberate terrorist attack, the emergency services may initiate a decontamination process if a large number of people are potentially contaminated. There are various methods of decontamination and in this study we are interested in what members of the public think about these different methods, and how they will experience these methods in real emergencies. The outcomes of this study will help inform the response of the police, fire and ambulance service.

Who can volunteer for the study?

We are looking for male and female participants aged over 18 to take part in the study.

Where and when will the study take place?

The study will take place at the King’s College London Denmark Hill campus in November 2015. If you decide to take part in the study, you will be asked to attend this site to take part in a focus group, which will last for approximately 2 hours. You will receive £60 in high street gift vouchers (redeemable at a large range of shops, restaurants, and attractions) to thank you for helping out.

**Do I have to take part?**

No. Participation in this study is entirely voluntary and you are free to withdraw at any time without giving a reason, with no penalty. If at any point during the focus group you wish to withdraw from the study, please inform the facilitator and you will be able to leave immediately and your data will not be used in the write-up of the study. If after the focus group you wish to withdraw, please inform a member of the research team within two weeks of taking part in the focus group, before your data is included in the analysis.

[
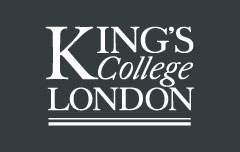
](http://www.google.co.uk/url?sa=i&rct=j&q=&esrc=s&frm=1&source=images&cd=&cad=rja&uact=8&ved=0CAcQjRxqFQoTCLPW5si-v8gCFYpAFAod5hAJpw&url=http://www.kcl.ac.uk/prospectus/graduate/master-of-laws&psig=AFQjCNEQClsKei63zmw18YTkEvJmj5OTjw&ust=1444826887670530)

**Further Information**

**What will happen on the day?**

On the day of the study you will arrive at the King’s College London Denmark Hill main reception and will be met by a member of the research team. You will be asked to sign a consent form to take part in the research.

Once you have signed a consent form, you will be asked to read a short scenario of a hypothetical emergency incident, and asked to visualise that you have been involved in the incident described. Following this, you will be asked to complete a short questionnaire. You will then take part in a focus group of between 6 – 8 people to discuss the scenario you have read. Following this, you will be asked to complete another short questionnaire, before being provided with more information about the nature of the incident described in the study.

What happens after the study?

Following the study, you will be given a debriefing statement containing more information about the research. You will also be given the opportunity to ask any questions which you may have. The debriefing statement will contain contact details of the research team, should you have any further questions after taking part in the research. You will also be sent a short questionnaire by email 3 months after the study.

#### Are there any risks for my health or well-being?

Staff members will be on hand at all times to help you if you become concerned. If you have any concerns about taking part prior to the study, please discuss them with Dr Richard Amlôt (contact details on page 3).

If you have taken part and feel that participation in this study has harmed you in any way, you can contact King's College London for further advice and information, using the details on Page 3.

If you would like more information about help and support which is available following an incident, or would just like to talk to someone about your experiences, please follow the link below:

<http://www.mind.org.uk/information-support/types-of-mental-health-problems/post-traumatic-stress-disorder-ptsd/about-ptsd.aspx>

**What are the possible benefits of taking part?**

Your participation in this study will enhance the emergency services ability to manage an incident involving the need for decontamination, should one occur. You may request a copy of the research findings following the study.

**Will my taking part in this study be kept confidential?**

The data you provide during this study will be kept confidential and anonymous in accordance with the 1998 Data Protection Act. At no point will any data be associated with your name or identity.

How will my information be stored/ used in the future?

Your data will be retained for 20 years after the study. At no point will you be identified. Your data will be held confidentially, with access restricted to researchers working in Behavioural Science, Public Health England. Once your data has been analysed, it may be used in publications in academic journals and reports. It may be presented to a variety of academic and professional audiences but you will not be identified.

[
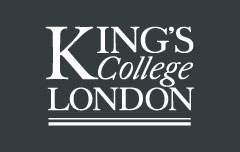
](http://www.google.co.uk/url?sa=i&rct=j&q=&esrc=s&frm=1&source=images&cd=&cad=rja&uact=8&ved=0CAcQjRxqFQoTCLPW5si-v8gCFYpAFAod5hAJpw&url=http://www.kcl.ac.uk/prospectus/graduate/master-of-laws&psig=AFQjCNEQClsKei63zmw18YTkEvJmj5OTjw&ust=1444826887670530)

Once the data has been analysed, it will not be possible to withdraw, however, if you wish to have your data deleted prior to analysis for any reason, please contact the researchers and, wherever possible, your request will be obliged.

Who is funding this study?

This study is funded by the National Institute for Health Research (NIHR), although the research itself is entirely independent of all our partner organisations.

Who is carrying out this study?

This study is carried out by Public Health England in collaboration with King’s College London and Newcastle University.

**Who has reviewed this study?**

An ethical review of this study has been carried out by the *Psychiatry, Nursing and Midwifery Research Ethics Committee [ref:* 14/15-1909*].*

**Contact for Further Information:**

For further information about this study, please contact Dr Richard Amlôt:

Telephone: 01980 612917

Email: Richard.amlot@kcl.ac.uk

If you have a concern about any aspect of this study, you should ask to speak to the researcher on the number above, who will do their best to answer your questions. The researcher will be available to answer calls or emails Monday to Friday, 9.00am to 5.00pm.

If this study has harmed you in any way, you can contact the Chair of the Psychiatry, Nursing and Midwifery Research Ethics Committee at [rec@kcl.ac.uk](mailto:rec@kcl.ac.uk).

Thank you very much for taking the time to consider this research study.

**If you decide to take part you are still free to withdraw at any time without giving a reason.**
