# Supplementary material for: Public perceptions of emergency decontamination: Effects of intervention type and responder management strategy during a focus group study
Source: PLoS One. 2018 Apr 13;13(4):e0195922. doi: 10.1371/journal.pone.0195922 (PMC5898741; doi:10.1371/journal.pone.0195922)
Supplement: S8 Text — (DOC) [file pone.0195922.s008.doc]

**S8 Text**

**CONSENT FORM**

Name of Lead Researcher: Dr. Richard Amlôt

Please initial all boxes

1. I confirm that I have read and understand the study information sheet dated [v1.1, 21/0915]. I have had the opportunity to consider the information, ask questions and have had these answered satisfactorily.

1. I understand that my participation is voluntary and that I am free to withdraw from the study at any time without giving any reason, without my legal rights being affected.
2. I understand that any information collected during this study will be held confidentially, in accordance with the Data Protection Act.
3. I understand that during the study, filming and audio recording will take place. I give permission for filming and audio recordings to be taken of me during the study. I understand that video and audio recordings will be used for research purposes only.
4. I understand that the outcomes of this study may be published in reports and journals, and that individuals participating in the study will not be identified in any of these reports.
5. I understand that due to the interactive and interdependent nature of focus groups, the absolute confidentiality of my contribution cannot be guaranteed.
6. I agree to take part in the above study.

Name of Volunteer Date Signature

Name of staff member taking consent Date Signature
